# Diplatin, a Novel and Low-Toxicity Anti-Lung Cancer Platinum Complex, Activation of Cell Death in Tumors *via* a ROS/JNK/p53-Dependent Pathway, and a Low Rate of Acquired Treatment Resistance

**DOI:** 10.3389/fphar.2019.00982

**Published:** 2019-09-11

**Authors:** Xixi Lin, Yongliang Jia, Xinwei Dong, Jian Shen, Yachao Jin, Yanyou Li, Fang Wang, Eitan Anenberg, Jiancang Zhou, Jianping Zhu, Xiaoping Chen, Qiangmin Xie, Yicheng Xie

**Affiliations:** ^1^Children’s Hospital, Zhejiang University School of Medicine, Hangzhou, China; ^2^Zhejiang Respiratory Drugs Research Laboratory of Food and Drug Administration of China, Zhejiang University School of Medicine, Hangzhou, China; ^3^Breath Smooth Biotech Hangzhou Co., LTD, Hangzhou, China; ^4^Beijing Shuobai Pharmaceutical Co., LTD, Beijing, China; ^5^Joinn Laboratories, BAD, Beijing, China; ^6^Sir Run Run Shaw Hospital, Zhejiang University School of Medicine, Hangzhou, China

**Keywords:** lung cancer, platinum complex, water solubility, ROS/p53 pathway, cisplatin resistance

## Abstract

**Background:** Platinum-based drugs prevail as the main treatment of lung cancer; this is caused by their relative effectiveness despite known side effects, such as neurotoxicity. The risk reward of the treatment and side effects is confronted when dosage is considered and when resistance to treatment develops. Development of new compounds that improve effectiveness and safety profiles addresses this ongoing need in clinical practice.

**Objectives:** The novel water-soluble platinum complex, diplatin, was synthesized, and its antitumor potency and toxicology profile were evaluated in murine xenograft tumor models and in lung cancer cell lines.

**Methods:** The effects of diplatin, cisplatin (DDP), and carboplatin (CBP) on the viability of nine lung tumor cell lines and one normal human lung epithelial cell line were evaluated using the MTT assay. Therapeutic index was calculated as LD_50_/ED_50_ to identify and compare the ideal therapeutic windows of the above compounds. Diplatin’s antitumor effects were assessed in lung xenograft tumors of nude mice; molecular mechanisms of therapeutic effects were identified.

**Results:** Diplatin had desirable IC_50_ compared to CBP in a variety of cultured tumor cells, notably lung tumor cells. In the mouse xenograft lung tumor, diplatin led to a substantially improved therapeutic index when compared to the effects of DDP and CBP. Importantly, diplatin inhibited the growth of DDP-resistant lung tumor cells. Diplatin’s mode of action was characterized to be through cell cycle arrest in the G2/M phase and induction of lung tumor apoptosis *via* ROS/JNK/p53-mediated pathways.

**Conclusion:** Diplatin was observed to have antitumor effects in mice with both greater potency and safety compared with DDP and CBP. These observations indicate that diplatin is promising as a potential treatment in future clinical applications.

## Introduction

Immense efforts are being made to prevent and treat lung cancer while cancer morbidity and mortality are ongoing (Hirsch et al., 2017). Relative to other forms of cancer, the survival rate in those with lung cancer is very low (Vachani et al., 2017). Current treatment approaches are based on the use of platinum-based drugs, such as cisplatin (DDP) and carboplatin (CBP), these compounds lesion the DNA of cancer cells effectively (Muggia et al., 2014; Carenco et al., 2015), but their practical clinical application is limited by severe toxicity. DDP can induce nephrotoxicity, neurotoxicity, and ototoxicity, whereas CBP causes myelosuppression, hepatotoxicity, and gastrointestinal side effects (Olaussen et al., 2006). Resistance to these drugs can be acquired (Galanski and Keppler, 2007), and toxicity can limit the doses that can be administered. The clinical effectiveness of platinum-based therapeutics is limited by toxicity and the potential for the targeted cells to develop resistance.

Advancements in approaches outside the scope of platinum-based therapy for improving clinical outcome of lung cancer patients exist; there is growing evidence that combining chemotherapies with immunotherapeutics is effective while circumventing lung cancer drug resistance. An example of such an approach, used in an effort to avoid harm of side effects from platinum-based drugs and chemotherapy, is the aerosol administration of both DDP (chemotherapy) and nivolumab (immunotherapy), which exert synergistic effects, and is an approach that is considered in the treatment of lung cancer (Sapalidis et al., 2018). Another example of an approach being developed for a similar clinical need is the use of oncolytic adenovirus and temozolomide, which has shown promise in cancer cell lines and mouse models (Gomez-Gutierrez et al., 2016). DDP when delivered in nanoparticles showed greater effectiveness in the suppression of lung tumor growth and increased survival in mice when compared to those treated with free DDP (Shi et al., 2015). Modification of platinum-based compounds is another promising approach to overcome the latter limitation, for example, with the creation of non-cross-resistant analogs of DDP (Zhao et al., 2016). However, addressing toxicity remains critical in translating newly developed compounds to clinical practice. Increasing the water solubility of platinum antitumor drugs has been an important practical objective of many drug development programs (Montana and Batalla, 2009; Liu et al., 2011), with the potential for greater water solubility of platinum drugs to reduce side effects, particularly in the case of nephrotoxicity. Water-soluble platinum complexes, which are relatively stable (slow hydrolysis), remain in the blood longer, and can be efficiently excreted intact *via* the kidneys. This approach has the potential for a reduced effective dose and an avoidance of toxic heavy metal accumulation in the body (Liu et al., 2013). One major approach to achieve this greater solubility is to alternate the chloride anions of DDP to appropriate leaving groups (e.g., nedaplatin). However, nedaplatin is a DDP analog with two amine ligands, like CBP, which is confirmed to be cross-resistant with DDP (Liu et al., 2007; Shimada et al., 2013). On the basis of these facts, synthesis of a novel platinum complex was motivated by modifying the non-leaving (di)amine ligand(s) to overcome cross-resistance of DDP in addition to the leaving group.

We recently synthesized diplatin, 2-(4-(diethyl-amino)butyl)malonate-O,O’]-[(1R,2R)-cyclohexane-1,2-diamine-N,N’] platinum (II) phosphate, where the addition of a malonic acid with amino as the leaving group substantially improved solubility and stability in water compared to DDP. Moreover, diplatin features a chelating R,R-diaminocyclohexane (DACH) non-leaving group ligand, which has long been investigated as a component in platinum anticancer agents (Johnstone et al., 2014). Here, we conduct an extended preclinical characterization of diplatin. We confirmed that diplatin exhibited reduced toxicity in mice compared to DDP and CBP. Its pharmacokinetic profile in dogs was comparable to that of DDP. Importantly, diplatin was effective against a broad spectrum of cancers, as observed in both cultured tumor cells and in mouse lung tumor xenografts. Here, we comprehensively characterized the effect of diplatin on lung cancer in a comparison to commonly used platinum drugs. We report quantitative measures of antitumor effects, off-target toxicology, pharmacokinetics, and characterized biochemical mechanisms of action.

## Materials and Methods

### Reagents

The diplatin used was a white fine powder with the purity of 99.9% produced by Beijing Shuobai Pharmaceutical Co., LTD. The DDP used was produced by Hospira Australia Pty Ltd. (Lot No. Y101881AB). The CBP used was produced by Bristol–Myers Squibb S.r.l. (Lot No. 0D57101). MTT (Lot No. M2128), NAC (Lot No. A7250), and DCFH-DA (Lot No. D6883) were ordered from Sigma-Aldrich (St. Louis, MO, USA). RPMI 1640, Glucose-DMEM, FBS, penicillin, and streptomycin were obtained from Thermo Fisher Scientific (Kalamazoo, MI). Antibodies against p-JNK (T183) (#4668), JNK (#9552), and β-actin (#3700) were acquired from Cell Signaling Technology (Beverly, MA), and p53 (BS1913), Fas (BS1745), Bax (BS6420), and VEGF (BS5540) were from Bioworld (Minnesota, USA). Lipofectamine 2000 (Lot No. 11668) was from Invitrogen (Carlsbad, USA). Cell cycle staining solutions (Lot No. 70CCS012) were purchased from Multi Sciences Biotech Co., Ltd. EdU cell proliferation kit (Lot No. C10310) was from RiboBio (Guangzhou, China), and the Annexin V–FITC apoptosis detection kit (Lot No. V13241) was from Invitrogen™ (Oregon, USA).

### Animals

Nude BALB/c mice and Sprague-Dawley rats (certificate no. SCXK2012-0002) from SLAC Laboratory Animal Co., Ltd. (Shanghai, China) and ICR mice (certificate no. SCXK 2012-0001) from Weitong-Lihua Experimental Animal Center (Beijing, China) were housed in Plexiglas cages, kept on a 12/12-h light–dark cycle, and received food and water *ad libitum* in a temperature- and humidity-controlled environment. All experimental procedures involving animals were performed in accordance with the Guide for the Care and Use of Laboratory Animals of Zhejiang University (Permit No. ZJU20170013) and were performed according to Institutional Animal Care and Use Committee based on guidelines from the National Institutes of Health.

### Cell Culture

All the cells used in the study were sourced from the Cell Bank of Chinese Academy of Sciences (Shanghai, China). The human cell lines used in the study have been compared and cross-checked with the STR profile database. These cells were grown in RPMI 1640 or Glucose-DMEM supplemented with 1.5 mg/ml sodium bicarbonate, 4.5 mg/ml glucose, 10 mM HEPES buffer, 10% fetal bovine serum (FBS), and 1% penicillin/streptomycin and incubated at 37°C in a humidified atmosphere containing 5% CO_2_.

### Assessment of Compound Water Solubility

The water solubility of diplatin was determined according to the 2015 edition of *Chinese Pharmacopoeia*. Diplatin was weighed and placed in the water of a certain capacity at 25 ± 2 °C, shaken vigorously for 30 s every 5 min, and watched for 30 min. The term “Very soluble” was assigned when 1 g solute can be dissolved in less than 1 ml solvent. “Soluble” was assigned when 1 g solute can be dissolved in 1 to 10 ml solvent. “Dissolution” was assigned when 1 g solute can be dissolved in 10 to 30 ml solvent. “Sparingly soluble” was assigned when 1 g solute can be dissolved in 30 to less than 100 ml solvent. “Slightly soluble” was assigned when 1 g solute can be dissolved in 100 to 1,000 ml solvent. “Very slightly soluble” was assigned when 1 g solute can be dissolved in 1,000 to less than 10,000 ml solvent. “Almost insoluble or insoluble” was assigned when 1 g solute cannot be completely dissolved in 10,000 ml solvent.

### Determination of IC_50_

The effects of diplatin, DDP, and CBP on the viability of the cells were measured using the MTT assay. Cells were seeded at a density of 1 × 10^4^ cells per well in 96-well plates. The following day, these cells were washed with PBS and exposed to different concentrations of testing compounds in culture medium for 48 h. MTT solution (10 μl at 5 mg/ml) was added to each well. After 4 h of incubation at 37°C, the medium was removed, and the insoluble precipitate was dissolved in 100 μl DMSO (Sigma, St. Louis, MO, USA). The absorbance of the dissolved solution at 490 nm was measured using a microplate reader (TECAN A-5082; Megllan, Austria). Cell viability percentages were calculated by dividing the mean optical density (OD) of compound-containing wells by that of the control wells. Each experiment was replicated three times. The IC_50_ of testing drugs was calculated with Statistical Product and Service Solutions software (SPSS, version 16.0).

### EdU Incorporation Assay

Cell proliferation was analyzed by measurement of DNA synthesis as identified by the EdU cell proliferation assay, which was applied in accordance with product manufacturer instructions. Cells (5 × 10^4^ cells per well) were cultured in triplicate in 24-well plates and treated with testing compounds for 48 h. Then the cells were incubated with 50 μM EdU for an additional 2 h at 37°C. Cells were fixed with 4% formaldehyde for 30 min and were permeabilized with 0.5% Triton X-100 for 10 min at room temperature. After washing with PBS three times, cells were incubated for 30 min with 1× Apollo reaction cocktail. Finally, cells were stained with 10 μg/ml of Hoechst 33342 for 30 min and then imaged using a confocal laser scanning microscope (FV1000; Olympus, Japan). The percentage of EdU^+^ cells was quantified using ImageJ software by dividing the mean OD of EdU^+^ cells to the mean OD of Hoechst-labeled cells.

### Cell Cycle Measurements

Cells (2 × 10^5^) were cultured in 6-well plates and exposed to diplatin, DDP, and CBP for 48 h. Then, the cells were trypsinized, washed with PBS, and fixed in 1.5 ml 95% ethanol and left at 4°C overnight. They were then incubated with RNase and stained with propidium iodide (PI) (Multi Sciences Biotech Co., Ltd). PI absorbance was determined using a Cytomics FC500 Flow Cytometer (Beckman Coulter, USA).

### Detection of Apoptosis

Following a similar preparation as described above for cell cycle measurements, cells in the early and late stages of apoptosis were detected using an Annexin V–FITC apoptosis detection kit. Cells (2 × 10^5^) were cultured in 6-well plates and exposed to each of the testing compounds for 48 h. Cells were identified as being in early apoptosis if positive for Annexin V–FITC alone and in late apoptosis if positive for both Annexin V–FITC and PI. Cells were counted using a Cytomics FC500 Flow Cytometer (Beckman Coulter, USA).

### Levels of Intracellular Reactive Oxygen Species

2′,7′-Dichlorofluorescein diacetate (DCFH-DA) was used to monitor intracellular reactive oxygen species (ROS) levels. Cells were plated in 6-well plates at a density of 2 × 10^5^ and were exposed to 0 μM and 25 μM diplatin for 48 h. DCFH-DA was added to the treated cells at a final concentration of 20 μM. Then, the cells were incubated for 30 min at 37°C. Fluorescence intensity was measured using a Cytomics FC500 Flow Cytometer (Beckman Coulter, USA) at an excitation wavelength of 488 nm and a detection wavelength of 525 nm.

### p53 siRNA Preparation and Transfection

Cells were cultured in 6-well plates for 24 h. Then, specific siRNA or scramble siRNA was transfected into A549 and H292 cells using lipofectamine 2000 according to the manufacturer’s instructions. Forty-eight hours after transfection, immunoblotting was performed to examine p53 silenced by siRNA. The p53-specific siRNA was purchased from GenePharma (Shanghai, China).

### Western Blot Analysis

Cells were treated with diplatin, DDP, and CBP before being lysed. After centrifugation at 12,000 rpm for 10 min, an equal amount of protein from each group was subjected to electrophoresis on 10% SDS-PAGE gels. The proteins in the gel were then electroblotted onto polyvinylidene fluoride (PVDF) membranes (Millipore, Massachusetts, USA). After incubation in the blocking buffer (1× TBS, 0.1% Tween-20, and 5% w/v dry nonfat milk) for 1 h at room temperature, the membranes were incubated with the primary antibody at 4°C overnight. Next, membranes were rinsed with TBST, and then secondary antibodies were applied (LI-COR, USA) at room temperature for 1 h. Immunoreactive bands were visualized by a two-color infrared imaging system (Odyssey, LI-COR, USA).

### Animal Tumor Models and Treatment

Male nude BALB/c mice (4–6 weeks) were used for xenograft models through subcutaneous implantation of A549, H292, and A549/DDP cells into the right forelimb armpit. Cells in logarithmic growth were washed with PBS and suspended in serum-free medium. Portions of the suspension (5 × 10^6^ cells in 0.1 ml/mouse) were mixed at a 3:1 ratio with Matrigel basement membrane matrix and injected subcutaneously into the mice. Following 2 weeks of growth, tumor tissues were cut into multiple 2 × 2 × 2 mm^3^ pieces and implanted using a range trocar subcutaneously (s.c. injection) into the left forepaw armpit of each mouse (Jiang et al., 2017). Treatment with pharmacology was started when the tumors reached an average volume of 100–200 mm^3^. The mice were randomly divided into six groups (n = 14/group). Either vehicle control or each drug was administrated *via* tail vein injection (0.1 ml/10 g body weight) every 3 days: a) 5% glucose (400 μl/mouse); b), c), and d) diplatin (30, 60, or 120 mg/kg, respectively); e) DDP (6 mg/kg); f) CBP (60 mg/kg).

Body weight and tumor size were assessed every 3 days. Tumor size was measured using a caliper, the volumes were calculated according to the following standard formula: V = (L × W^2^)/2 (V = tumor volume, L = largest diameter of tumor, W = smallest diameter of tumor). On day 25, all mice were sacrificed by cervical dislocation, and the tumors were removed and weighed.

### Measurement of Biodistribution

To measure diplatin accumulation in tissues, A549 xenograft nude mice were intravenously administered with 120 mg/kg diplatin (equivalent to Pt 36.78mg/kg). At each time point of 10 min, 2 h, 6 h, and 24 h after administration, six mice were sacrificed. The tumors, organs/tissues, and blood were immediately collected. Then, Pt concentration was measured by atomic absorption spectrometry, counting the weight/volume of the tumors, tissues, organs, or blood.

### Determination of ED_50_ and LD_50_

To determine the dose that was effective for the reduction of 50% volume of the xenograft tumors (ED_50_), groups composed of A549, H292, and LTEP-A-2 xenograft nude mice (n = 14) were injected with the doses of diplatin at 4, 8, 16, 32, 60, and 120 mg/kg. Then, a total of 50 normal ICR mice (18–22 g), half male and half female, were used to determine LD_50_, where high doses of diplatin (200, 300, 400, 500, and 600 mg/kg) were administered by tail vein injection, each dose was tested on 10 mice (five male and five female). After injection, the animals’ condition was monitored every day for over 1 week by an observer who was blinded to the dose and drug allocation.

Estimates for ED_50_ and LD_50_ were calculated through nonlinear regression, data were fitted to a sigmoidal dose-response relationship in Prism (version 4.0a; GraphPad Software, Inc., San Diego, CA). The data were fitted to the equation:

$$Y=Y_{\min}+\frac{Y_{\max}-Y_{\min}}{1+10^{(Log{ED}_{50}-X)*HillSlope}}$$

where X is the logarithm of concentration, and Y is the response.

The mortality versus dose graph was made using DeltaGraph (version 5.6.1; SPSS Inc., Chicago, IL, and Red Rock Software, Inc., Salt Lake City, UT).

### TUNEL Assay

Tumor tissues were fixed in 10% formalin for 4 h and then embedded in paraffin. Five-micrometer-thick sections were sliced and stained for apoptotic cells with TUNEL assay kit in accordance with manufacturer instructions (KGI Biotechnology Company, Nanjing, China). A positive result was marked by brown staining in the cell nucleus. Cell images were acquired through a microscope (Olympus BX 51, Japan). The apoptotic index (AI) was determined as a percentage of apoptotic cells from at least 1,000 cells in each mouse.

### Immunohistochemistry

All tumor tissues were harvested and fixed in 4% buffered formalin. After 72 h, tumor tissues were embedded in paraffin and cut into 4 μm sections for immunohistochemistry assays. The streptavidin–biotin complex (SABC) method was used for immunohistochemical staining. Lung tissue sections from mice with no intervention were used as controls. The section slides were baked in an oven at 60°C for 1 h, washed in xylene, and hydrated in different concentrations of alcohol. Subsequently, endogenous peroxidase activity was quenched with 3% hydrogen peroxide for 10 min. To unmask the antigen, the slides were submerged in citrate buffer (0.01 M, pH 6.0) at 95°C for 5 min. The slides were blocked for 20 min with normal goat serum blocking solution. Primary antibodies were incubated at 4°C overnight. Primary antibody dilutions were as follows: 1:200 for VEGF, p53, and Bax. Immunocomplexes were visualized with 3,3′-diaminobenzidine (DAB), and the density was measured with the DP2-BSW software (Olympus, Tokyo, Japan).

### Data Analysis

Results are presented in figures as the mean ± SEM. One-way analysis of variance (ANOVA), followed by the Student-Newman-Keuls test, was used to determine multiple comparisons. Significant differences were defined at *P* < 0.05. Statistical analyses were conducted with SPSS software (version 16.0; SPSS, Chicago, IL).

## Results

### Diplatin Safety and Effectivity Profile

The LD_50_ and ED_50_ of diplatin, DDP, and CBP were determined as summarized in **Table 1**. Diplatin had a greater LD_50_ (496 mg/kg) compared to both DDP (13 mg/kg) and CBP (150 mg/kg). Moreover, diplatin offered significant beneficial outcomes in suppressing A549, H292, and LTEP-A-2 xenograft tumors with an average ED_50_ at 52 mg/kg, which was comparable to that of CBP, and nearly 10-fold larger than that of DDP. Therapeutic index (TI) is the ratio of LD_50_ to ED_50_ for a drug; a large TI indicates a wider window of safety for an effective treatment (Snider et al., 2016). We found the TI of diplatin, DDP, and CBP to be 9.53, 2.17, and 2.52, respectively, giving diplatin the largest window of safety.

**Table 1:** *In vivo* LD50 and ED50 for mice and calculated therapeutic index (LD50/ED50) of complexes.

Complexes	Diplatin	DDP	CBP
Mice LD50 (mg/kg)*	496	13.0	150
Mice ED50 (mg/kg)^★^	52.0	6.0	59.5
Therapeutic index	9.53	2.17	2.52

*LD50 in normal ICR mice.

^★^ED50 in A549, NCI-H292, NCI-H460, and LTEP-A-2 xenograft models.

Therapeutic index = LD50/ED50.

### The Cytotoxic Effects of Diplatin, DDP, and CBP Treatment on Lung Tumor Cell Lines

Next, we compared the cytotoxic effects of diplatin, DDP, and CBP on nine lung tumor cell lines and one normal human lung epithelial cell line, BEAS-2B (**Table 2**). Diplatin exerted potent effects against the lung tumor cell lines. The IC_50_ of diplatin ranged from 24.6 μM to 161 μM, which was significantly more potent than what was observed with CBP treatment (57–790.4 μM). Diplatin had threefold less suppression on the viability of BEAS-2B cells, a normal human lung epithelial cell line, compared to DDP. Diplatin partially overcame the DDP resistance in A549/DDP cells and showed nearly 2.5-fold better suppression of A549/DDP cells than CBP.

**Table 2:** The cytotoxic effects of diplatin, DDP, and CBP on lung cancer cell lines as determined by MTT.

Cell type	Cell line	IC_50_ (µM)		
		Diplatin	DDP	CBP
Lung	A549	102.0	46.7	220.6
	H292	30.9	6.0	256.7
	H460	40.7	10.3	228.8
	HCC827	25.0	14.7	328.1
	H1299	57.5	18.7	274.2
	LTEP-A-2	24.6	6.2	57.0
	H1650	36.6	11.6	208.8
	Calu-3	161.0	33.5	790.4
	A549/DDP	132.9	151.3	300.3
	BEAS-2B	47.9	19.3	460.6

### Diplatin Treatment Exhibits Potent Antitumor Activities Against Murine Models of Xenograft Lung Tumors With Low Toxicity

**Figure 1:**
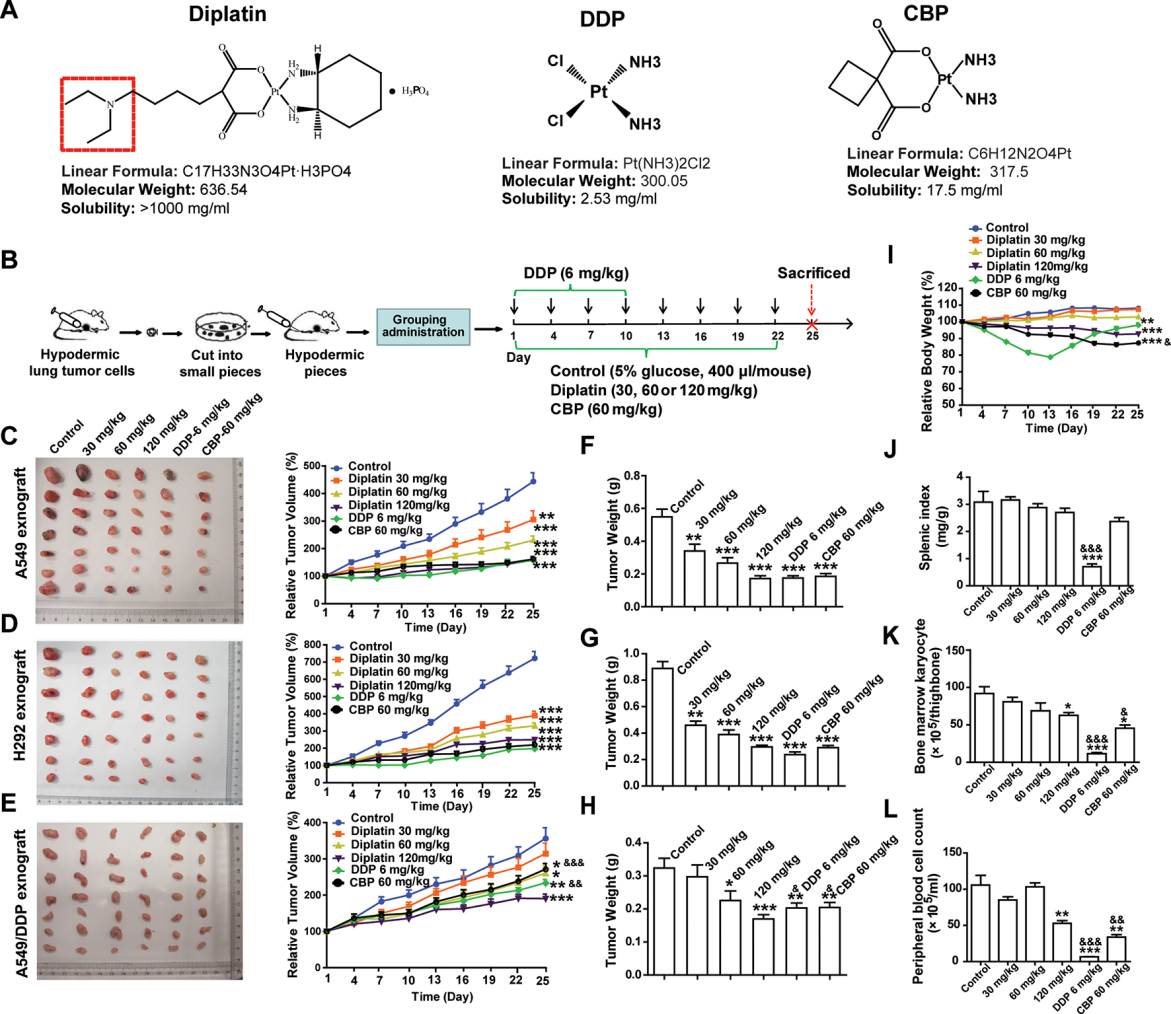
Effects of diplatin compared to other platinum-based pharmacology on xenograft tumor size. **(A)** Structure of studied platinum drugs: diplatin, DDP, and CBP. **(B)** Time course of the treatment (every 3 days *via* tail vein injection for 22 days of CBP and diplatin and only for 10 days of DDP because of its life-threatening toxic effects). Dose-dependent effects of diplatin (30, 60, 120 mg/kg), DDP (6 mg/kg), and CBP (60 mg/kg) on **(C)** A549, **(D)** H292, and **(E)** A549/DDP xenograft tumor growth relative to control (5% glucose). Tumor weight of **(F)** A549, **(G)** H292, and **(H)** A549/DDP in all treatment conditions compared with the control (taken from day 25). **(I)** Effects of diplatin (30, 60, 120 mg/kg), DDP (6 mg/kg), and CBP (60 mg/kg) on body weight. **(J)** Effects of diplatin (30, 60, 120 mg/kg), DDP (6 mg/kg), and CBP (60 mg/kg) on splenic index (spleen weight-to-body weight ratio). **(K)** Effects of diplatin (30, 60, 120 mg/kg), DDP (6 mg/kg), and CBP (60 mg/kg) on bone marrow karyocyte count. **(L)** Effects of diplatin (120 mg/kg), DDP (6 mg/kg), and CBP (60 mg/kg) on peripheral blood cell count. Group data are presented as the mean ± SEM (n = 14 per group), one-way ANOVA followed by the Student-Newman-Keuls test. *p < 0.05, **p < 0.01, and ***p < 0.001 compared with the control, ^&^ *p* < 0.05, ^&&^ *p* < 0.01 and ^&&&^ *p* < 0.001 compared with the 120 mg/kg diplatin–treated group.

DDP (chloro leaving ligands, considerably reactive, fast hydrolysis) is equipped with a severe renal toxicity. CBP, having a more stable dicarboxylato ligand (slow hydrolysis), exhibits an altered toxicological profile. Here, a novel water-soluble platinum complex, diplatin, was synthesized through replacing the oxalic acid ligand of oxaliplatin with a malonic acid attached *via* a tertiary amine group (red dotted box in **Figure 1A**). Because the oxalic acid ligand was replaced by a malonic acid, which does not precipitate with calcium ions, a significant source causing neurotoxicity, the new compound could theoretically avoid the neurotoxicity that is associated with oxaliplatin (Marmiroli et al., 2015). Accordingly, diplatin was determined to be very soluble in water (>1,000 mg/ml), 1 g diplatin was completely dissolved in 0.9 ml water. This was nearly 50, 200, and almost 400 times more soluble than CBP (17.5 mg/ml), oxaliplatin (6.1 mg/ml), and DDP (2.53 mg/ml), respectively.

A549, H292, and A549/DDP xenograft tumor models were established in nude mice (**Figure 1B**). Diplatin-treated group showed significant inhibition of xenograft tumor growth. Diplatin dose-dependently inhibited the growth of A549 xenograft tumors (**Figure 1C**). With 120 mg/kg, the inhibitory effects of diplatin on A549 xenograft tumor growth were comparable to that of DDP at 6 mg/kg and CBP at 60 mg/kg. Diplatin at 120 mg/kg significantly inhibited H292 xenograft tumor growth by 65.0% (*P* < 0.001); that was comparable to the 6 mg/kg DDP and 60 mg/kg CBP treatment (**Figure 1D**). Moreover, diplatin at 120 mg/kg showed more potent antitumor activities than DDP and CBP in A549/DDP xenograft tumor (**Figure 1E**). The antitumor effects of diplatin on another xenograft lung tumor, LTEP-A-2, were also assessed. The diplatin-treated group showed a significant inhibition on xenograft tumor volume and weight, which were comparable to the effects of DDP at 6 mg/kg and CBP at 60 mg/kg (**Supplementary Figure 2**).

The A549 and H292 xenograft mice were weighed every 3 days. Diplatin at 30 mg/kg and 60 mg/kg had no observable effects on the body weight. Diplatin at 120 mg/kg reduced animal weight by 14.38% (*P* < 0.001) by day 25 (**Figure 1I**). Body weight of the 60 mg/kg CBP treatment group dropped significantly more (18.71% of the baseline, *P* < 0.001). DDP had substantial toxic effects, which nearly caused the death of the mice, leading us to stop DDP administration after 10 days of treatment.

On day 25, all mice were killed, and tumors were weighed. The average weights of A549, H292, and A549/DDP xenograft tumors when treated with diplatin were reduced in a pattern that appeared to be dose-dependent. Diplatin at 120 mg/kg exhibited potent antitumor effects on reduction of tumor weight, which were similar to that of 6 mg/kg DDP and 60 mg/kg CBP treatment in A549 and H292 xenograft tumors (**Figure 1F, G**). This treatment was more potent than DDP at 6 mg/kg and CBP at 60 mg/kg in A549/DDP xenograft tumor (**Figure 1H**).

We measured diplatin accumulation in various tissues. We found large amounts of diplatin in the kidneys compared to the other organs (**Supplementary Figure 1A**). Pt accumulation was 2.2 times higher in the kidneys in the diplatin-treated group at 6 h after administration compared to CBP given at 70 mg/kg (equivalent Pt dose to 120 mg/kg diplatin, **Supplementary Figure 1B**). There was higher area under concentration time curve (AUC) in the mouse kidney 6 h after being given the 120 mg/kg diplatin treatment compared to those in the other organs or the blood (**Supplementary Figure 1C**).

To determine whether diplatin treatment caused any adverse effects to organs or hematopoiesis, we collected bone marrow, splenic, kidney, and peripheral blood from each animal. The splenic index, spleen weight-to-body weight ratio, was significantly lower in the DDP-treated group (**Figure 1J**), whereas the diplatin- and CBP-treated groups had no difference relative to control. Compared with the 120 mg/kg diplatin-treated group, DDP and CBP decreased bone marrow karyocyte count more (**Figure 1K**). Although 120 mg/kg diplatin treatment significantly decreased the peripheral blood cell count (50.0%, *P* < 0.01), the DDP and CBP group reduced the count more, which dropped by 93.8% (*P* < 0.001) and 67.9% (*P* < 0.01), respectively (**Figure 1L**). We monitored behaviors typically associated with neurotoxicity. There were no notable changes in the behavior (altered nesting) and activity (altered exploring) of the mice treated with diplatin at the dose of 60, 120, or 240 mg/kg twice a week for 4 weeks. In one and two out of eight mice, there was a slight change in appearance (e.g., kyphosis and altered grooming, respectively) but only in the group treated with 240 mg/kg (**Supplementary Table 3**). Given diplatin accumulated in the kidneys. It was not surprising that the diplatin treatment (7.5, 15, and 30 mg/kg twice a week for 4 weeks) resulted in nephrotoxicity in the testing rats; they showed renal epithelial cysts, interstitial fibrosis, interstitial fibroblast proliferation, tubular epithelium denaturation/regeneration, and renal tubular dilation (**Supplementary Table 4**), which was not greater than other tested platinum-based antitumor drugs. The doses of diplatin used had greater antitumor potency and lower systemic toxicity in most measures when compared with those of DDP and CBP.

### Diplatin Treatment Suppresses DNA Replication and Induces Cell Cycle Arrest in Lung Tumor Cells

**Figure 2:**
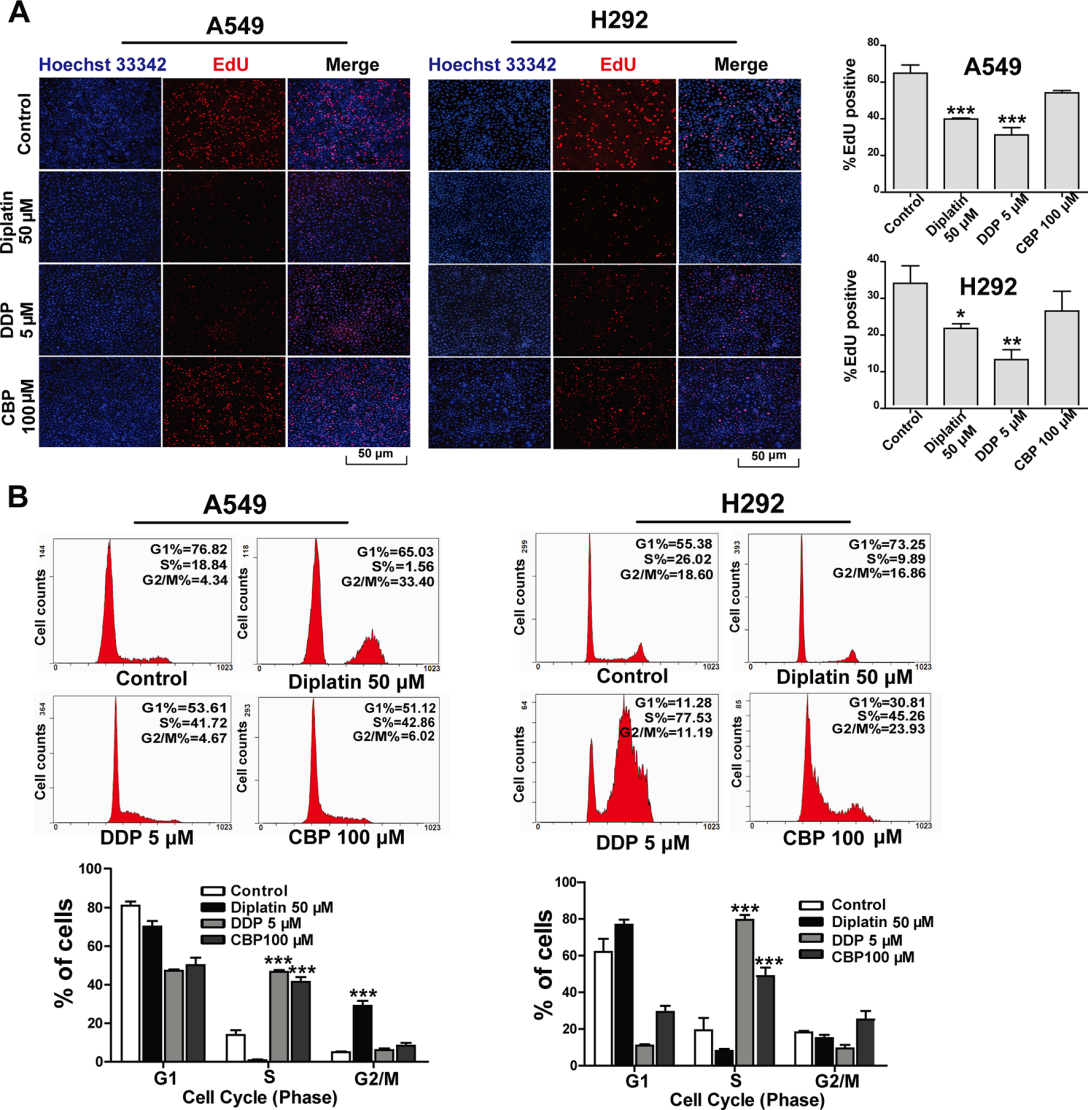
Effects of diplatin compared to other platinum-based pharmacology on cell proliferation in the lung tumor cells. **(A)** Forty-eight hours of exposure to diplatin, DDP, and CBP induced DNA replication in A549 and H292 cells. The EdU labeling (red) and the Hoechst 33342 labeling of DNA (blue) are shown. Group data are presented as the mean ± SEM (taken from three independent experiments). **(B)** Effects of diplatin (50 µM), DDP (5 µM), and CBP (100 µM) on cell cycle in A549 and H292 cells. Representative DNA fluorescence histograms of propidium iodide (PI)-stained cells (n = 4 per group). The data are shown as the mean ± SEM from three independent experiments, one-way ANOVA followed by the Student-Newman-Keuls test, *p < 0.05, **p < 0.01, and ***p < 0.001 compared to control.

EdU incorporation assay was employed to determine the impact of diplatin on DNA replication in the lung tumor cells. A549 and H292 cells were incubated with diplatin (50 μM), DDP (5 μM), and CBP (100 μM) for 48 h followed by EdU labeling. As shown in **Figure 2A**, EdU incorporation was dramatically decreased by diplatin and DDP treatment in A549 and H292 cells but not by CBP treatment. Diplatin appeared to be more potent than CBP in suppressing the initiation of DNA replication in lung tumor cells.

Flow cytometry was employed to detect the effects of diplatin on the distribution of cells in respective phases of their cycle. DDP and CBP treatment induced cell cycle arrest in S phase (**Figure 2B**). Diplatin 50 μM caused a significant accumulation of cells in G2/M phase in A549 cells (**Figure 2C**) and LTEP-A-2 cells (**Supplementary Figure 3**). In the case of H292 and H460 cell lines, diplatin failed to arrest any portion of the cell cycle (**Figure 2C** and **Supplementary Figure 3**). Despite the treatment working consistently in rodents, the effects of diplatin appear to differ across cell types.

### Diplatin Treatment Induces JNK/p53-Mediated Apoptosis in Lung Tumor Cells

**Figure 3:**
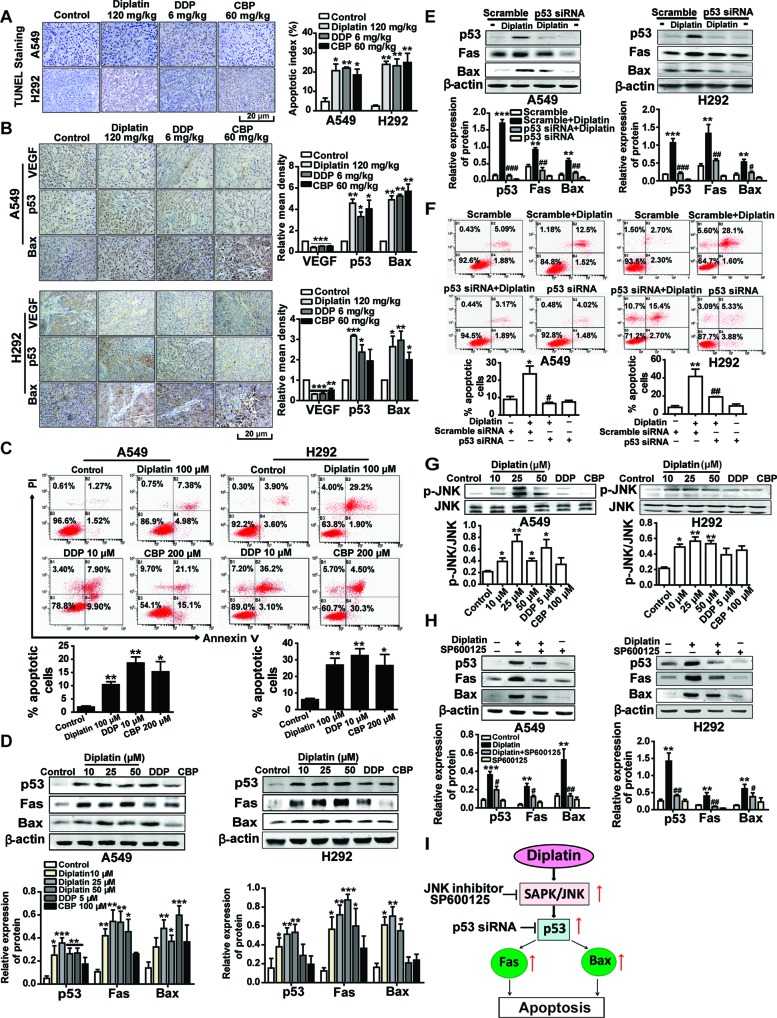
JNK/p53-mediated pathway is involved in diplatin-induced apoptosis of tumor cells. **(A)** TUNEL-stained sections of tumor cells after diplatin (120 mg/kg), DDP (6 mg/kg), and CBP (60 mg/kg) staining. Representative images from TUNEL staining are presented where apoptotic cells are indicated in dark brown. Apoptosis was quantified as the percentage of apoptotic cells (apoptotic index). Results are presented as the mean ± SEM from three independent experiments and are compared to control. **(B)** Immunohistochemical staining for VEGF, p53, and Bax expression in the xenograft A549 and H292 lung tumor tissue after treatment with diplatin (120 mg/kg), DDP (6 mg/kg), and CBP (60 mg/kg). Images are representative of three independent experiments. **(C)** Apoptotic effects of diplatin (100 μM), DDP (10 μM), and CBP (200 μM) exposure for 48 h on A549 and H292 cells, as measured by a flow cytometry-based apoptosis assay using Annexin V-FITC/PI double staining (n = 4 per group). **(D)** Effects of 48 h exposure of A549 and H292 cells to diplatin (10, 25, and 50 μM), DDP (5 μM), and CBP (100 μM) on p53, Fas, and Bax protein regulation as measured by western blot (n = 6 per group). **(E)** Consequence of p53 siRNA (100 nM) transfection in A549 and H292 cells on diplatin-induced Fas and Bax protein upregulation (n = 6 per group). The data are presented as the mean ± SEM from three independent experiments, one-way ANOVA followed by the Student-Newman-Keuls test. Statistical comparison of cells (scramble siRNA) without diplatin exposure (**p < 0.01, ***p < 0.001) compared with the cells (p53 siRNA) with diplatin exposure (^##^ *p* < 0.01 and ^###^ *p* < 0.001). **(F)** Transfection of A549 and H292 cells with p53 siRNA suppresses 100 μM diplatin-induced apoptosis, as measured by a flow cytometry-based apoptosis assay (n = 4 per group). The data are presented as mean ± SEM from three independent experiments, one-way ANOVA followed by the Student-Newman-Keuls test. **p < 0.01 comparison of cells (scramble siRNA) without diplatin exposure, ^#^ *p* < 0.05 and ^##^ *p* < 0.01 comparison of cells (p53 siRNA) with diplatin exposure. **(G)** Diplatin exposure at various concentrations for 0.5 h induces JNK phosphorylation in A549 and H292 cells measured by Western blot (n = 4 per group), with an apparent maximum at the dose of 25 μM. **(H)** Pretreatment (0.5 h) with 10 μM JNK inhibitor (SP600125) suppresses the 48 h time point 25 μM diplatin-induced p53, Fas, and Bax protein upregulation (n = 4 per group). The data are presented as mean ± SEM from three independent experiments, one-way ANOVA followed by the Student-Newman-Keuls test. Statistical significance is indicated by *p < 0.05, **p < 0.01, and ***p < 0.001 for comparison with control and ^#^ *p* < 0.05, ^##^ *p* < 0.01 for comparison with the diplatin-treated group. **(I)** A schematic of the apoptotic pathway induced by diplatin in lung tumor cells.

We next examined whether tumor inhibition by diplatin was a consequence of cell apoptosis through TUNEL staining and flow cytometry. Diplatin (120 mg/kg)-treated A549 and H292 tumors exhibited markedly increased apoptosis (20.7%, *P* < 0.05, and 23.8%, *P* < 0.01) that were comparable to the effects of the 6 mg/kg DDP (21.9%, *P* < 0.01, and 22.6%, *P* < 0.01) and 60 mg/kg CBP (18.5%, *P* < 0.05, and 24.9%, *P* < 0.01) treatment (**Figure 3A**). Correspondingly, both A549 and H292 cells exhibited significantly increased apoptosis following 100 μM diplatin exposure assessed by flow cytometry. These effects were comparable to 10 μM DDP treatment and were more effective than 200 μM CBP (**Figure 3C**). The effects of diplatin on tumor angiogenesis were observed by immunolabeling vascular endothelial growth factor (VEGF). Apoptosis in tumor tissue was measured by immunolabeling apoptosis-related proteins, p53 and Bax. The diplatin-treated group (120 mg/kg) showed lower expression of VEGF compared to the control (**Figure 3B**). Diplatin treatment significantly enhanced p53 and Bax expression in the A549 and H292 tumor sections. Effects of diplatin on p53 expression were nearly two-fold more potent than those of DDP and CBP treatment at the dose of 120 mg/kg. Next, following diplatin treatment *in vitro*, we assessed the expression of p53 and its downstream proteins. Our results showed that diplatin enhanced p53, Fas, and Bax expressions in A549 and H292 cells; these effects were approximately 1.5-fold more pronounced than those of DDP at 5 μM and CBP at 100 μM (**Figure 3D**).

We confirmed that p53 regulated Fas and Bax activation in response to diplatin treatment by using siRNA-mediated knockdown of p53 in A549 and H292 cells (**Figure 3E**) and observing that the antitumor activity of diplatin was p53-dependent (**Figure 3F**). We found that JNK phosphorylation levels, other than those of Erk1/2 or p38 (**Supplementary Figure 3**), were elevated following diplatin (10–50 μM) and 10 μM DDP treatment in A549 and H292 cells (**Figure 3G**). JNK inhibition by SP600125 suppressed diplatin-induced p53 (*P* < 0.05 and *P* < 0.01), Fas (*P* < 0.05 and *P* < 0.01), and Bax (*P* < 0.01 and *P* < 0.05) expression in the two cells (**Figure 3H**). JNK activation has a role in p53-mediated mitochondrial and death receptor apoptosis following diplatin treatment (**Figure 3I**).

### Diplatin Treatment Induces Lung Tumor Cell Apoptosis *via* a ROS-Dependent Mechanism

**Figure 4:**
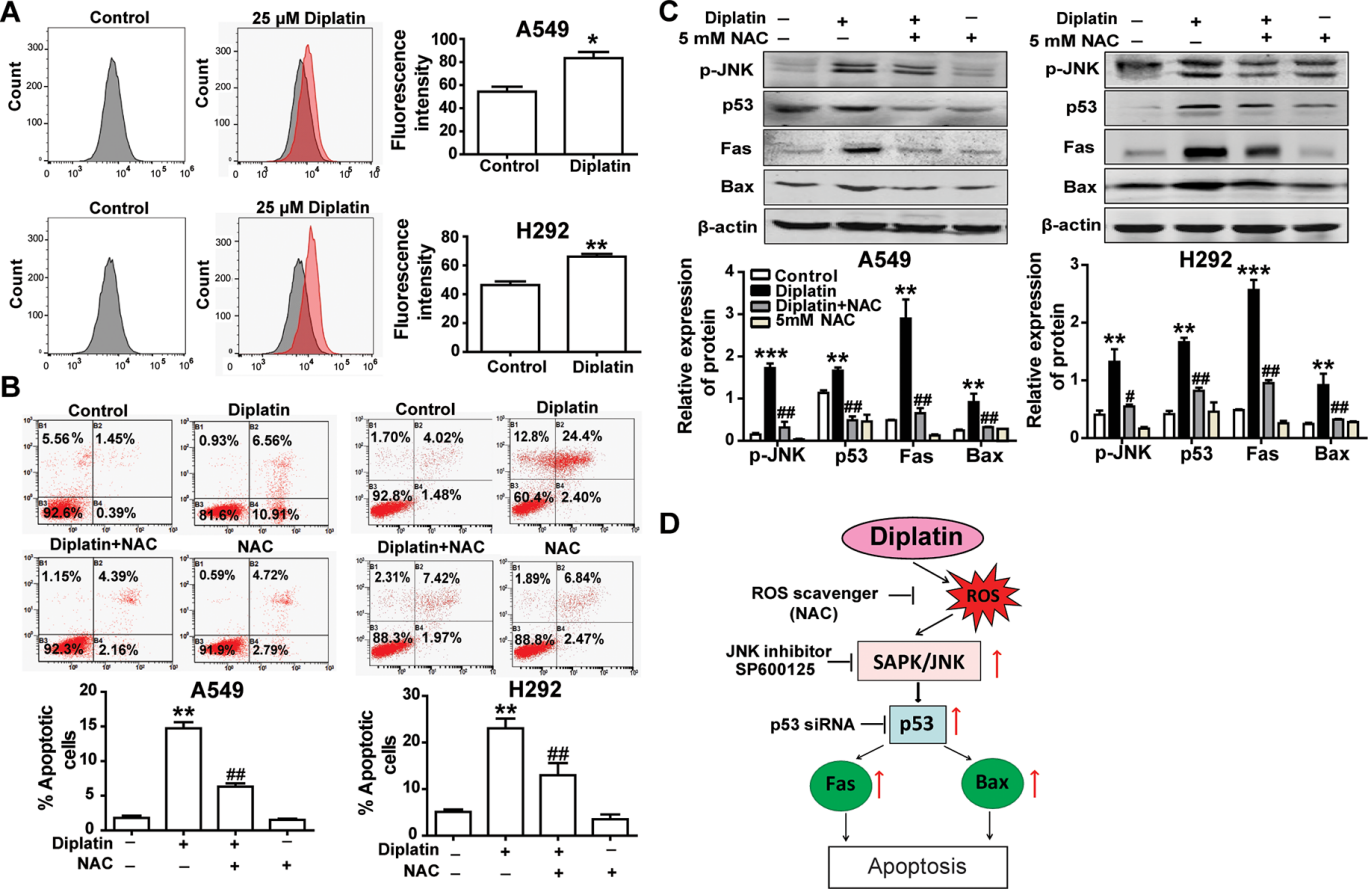
Diplatin treatment induces lung tumor cell apoptosis *via* a ROS-dependent pathway. **(A)** Diplatin induces a significant increase in ROS generation in A549 and H292 cells detected by flow cytometry-based assay using DCFH-DA. The data are presented as the mean ± SEM from three independent experiments. **(B)** Pre-treatment (0.5 h) with a ROS scavenger (NAC) suppresses 48 h the diplatin-induced (100 μM) apoptosis in A549 and H292 cells, as determined by the flow cytometry-based apoptosis assay (n = 4 per group). **(C)** NAC induces downregulation of p-JNK, p53, Fas, and Bax protein expression after pretreatment (0.5 h) with diplatin (25 μM) (n = 4 per group). The data represent the mean ± SEM from three independent experiments, one-way ANOVA followed by the Student-Newman-Keuls test, *p < 0.05, **p < 0.01, and ***p < 0.001 compared with the control, ^#^ *p* < 0.05, ^##^ *p* < 0.01 compared with the diplatin-treated group. **(D)** A schematic of ROS-dependent apoptotic mechanisms induced by diplatin treatment.

A549 and H292 cells treated with 25 μM diplatin showed nearly a 1.5-fold higher ROS production compared with the control (**Figure 4A**). Flow cytometric analysis showed that the diplatin-induced apoptosis was attenuated by NAC pretreatment (scavenging ROS production) (**Figure 4B**). Pretreating with NAC reduced the expression of cell death marker p-JNK, p53, Fas, and Bax, which were upregulated by diplatin (**Figure 4C**). This indicates that the diplatin-induced ROS triggered apoptosis upstream of the JNK–p53 pathway (**Figure 4D**).

## Discussion

In the present study, we reported the first anticancer application of a novel water-soluble platinum complex-diplatin effectively inhibited lung tumor growth with improved safety. Its antitumor potency was demonstrated in multiple murine models of xenograft tumors. In a comparison of effectiveness to commonly used platinum drugs, DDP and CBP, diplatin exhibited a superior therapeutic index (trade-off between effectiveness and safety). This indicates potential for successful clinical usage and a rationale for additional investigation. Interestingly, diplatin inhibited the growth of DDP-resistant lung tumor cells. Overcoming resistance while maintaining safe doses is a therapeutic challenge. Our data suggest that diplatin may have a role in therapy for addressing DDP-resistant cancer. We demonstrated that the effects of diplatin were from both the inhibition of proliferation and facilitation of apoptosis in lung tumor cells through ROS generation and enhanced activation of the JNK–p53-dependent cell death signaling pathway.

Non-small-cell lung cancers (NSCLC) encompass the majority of lung cancer incidence (Bahcall et al., 2018; Wu et al., 2018) and thus were examined in this study. We found that diplatin could have a broad spectrum of applications, also suppressing the growth of gastric cancer cells (**Supplementary Table 1**). In therapeutically tolerated doses, diplatin was five-fold more effective than CBP and as effective as DDP. A plethora of future work to comprehensively evaluate the effectiveness of diplatin in treating other tumor types remains.

Here, we found that diplatin exhibited a wider window of safety compared to DDP and CBP; animals having comparatively little weight loss and low toxicity to organs (kindey, spleen, and bone marrow). Mice treated with DDP (Wu et al., 2018) or CBP (Hess et al., 2007) were shown to become gradually less active with lower food intake and significant loss in body mass. Animals treated with comparably effective diplatin doses had none of these side effects or any significant toxicity, such as that observed with DDP treatment (**Supplementary Table 2**). In clinical practice, preserving weight during concurrent chemoradiation therapy is of great importance because it is strongly correlated with survival outcomes (Topkan et al., 2013). Reducing drug side effects is critical because intolerance to platinum drugs during treatment, namely, DDP, can lead to patients abandoning this therapy (Crona et al., 2017). DDP has poor pharmacokinetic properties; it binds off target to plasma proteins, spontaneously degrades in the bloodstream, and is rapidly cleared from the blood by glomerular excretion (Yu et al., 2015). It is also toxic, with urinary concentrations of DDP positively correlated with the degree of nephrotoxicity (Yin et al., 2015). Synthesis of diplatin was motivated by increasing the water solubility of DDP to overcome issues related to toxicity; this was done by modification of the leaving ligands of DDP (Liu et al., 2013). For DDP to be tolerated by the animals, we found a need to use lower than previously reported treatment doses (6 mg/kg, i.v. injection) in our treatment nude mice (Goto et al., 2004; Ren et al., 2014; Sen et al., 2017).

Diplatin induced apoptosis in lung tumor cells through JNK-activated p53 upregulation. The activation of p53 further upregulated Fas and Bax expression. JNK activation triggers apoptosis (Davis, 2000), along with upregulation of p53 expression and other downstream targets (Fuchs et al., 1998; Fan et al., 2001). p53 is a key player in the process of mitochondria-mediated apoptosis; it enhances the expression of Bax (Follis et al., 2015) and induces Fas upregulation. This is consistent with observations from chemotherapeutic drug treatments (Guo et al., 2014). Diplatin induced apoptosis through the elevation of intracellular ROS in the lung tumor cells. Diplatin also inhibited JNK phosphorylation and the subsequent expression of downstream proteins involved in cell death signaling (p53, Fas, and Bax). Here, we demonstrate that mechanisms of diplatin-induced apoptosis occur *via* increasing ROS levels that enhance the presence of proapoptotic signaling molecules (Shen and Liu, 2006; Shi et al., 2014).

Diplatin treatment at the indicated dose did not significantly alter the kidney index (data not shown); however, there was notable accumulation of diplatin in the kidneys of treated mice and the clear renal pathological changes in the diplatin-treated rats. This suggests the presence of some nephrotoxicity. Clinical application of DDP is in part limited by nephrotoxicity with a single dose of DDP (50–100 mg/m^2^), leading to approximately one-third of the patients developing nephrotoxicity (Ozkok and Edelstein, 2014). DDP-treated rats exhibited approximately five-fold higher Pt accumulation in the kidney than the CBP group (Moraleja et al., 2018), whereas Pt accumulation was 2.2 times higher in the kidneys of the diplatin-treated group relative to those of the CBP group (6 h after administration). Based on our results, we suggest that diplatin may exert more nephrotoxicity than CBP, but much less than DDP.

Previous studies have reported improved antineoplastic effects with platinum accumulation in tumors (Mostaghimi et al., 2017). We found that the Pt concentration in the diplatin-treated tumors was 1.7-fold higher than that of the CBP group, as assessed by atomic absorption spectrometry (**Supplementary Figure 1**). This indicates that the observed superior antitumor effects of diplatin relative to CBP from the administered doses led to the same Pt concentration. Diplatin induced cell cycle arrest in G_2_/M phase in A549 and LTEP-2-A cells. This is distinct from the effects of DDP and CBP, which arrest the cell cycle in S phase (Zhang et al., 2015). The G_2_/M checkpoint is when the cell can repair DNA damage before entering mitosis (Kastan and Bartek, 2004) and is the most conspicuous target for developing anticancer drugs that directly lesion DNA (Huang et al., 2011). DDP resistance in tumor cells is in part attributed to the loss of G2/M arrest (Horibe et al., 2015); diplatin arresting A549 and LETP-2-A cells in G2/M is a potential hypothesis for its ability to be effective in treating DDP-resistant tumor cells. Development of resistance to chemotherapy in NSCLC is accompanied by a decrease in the expression of Fas and FasL (Okouoyo et al., 2004), whereas upregulation of Fas expression can reverse the DDP resistance of human small-cell lung cancer and ovarian cancer (Wu et al., 2010; Yang et al., 2015). Diplatin, at a comparably effective dose to DDP and CBP, led to greater Fas expression. JNK phosphorylation, involved in cell apoptosis, is also reduced in tumor cells during DDP treatment as resistance is acquired (Brozovic et al., 2004). Diplatin, at a comparably effective dose (to CBP and DDP), led to greater induction of JNK phosphorylation. These mechanistic findings explain in part why diplatin was effective against DDP resistance in NSCLC cells.

Diplatin was found to have potent antitumor effects. Diplatin treatment could overcome DDP-resistant cells and was less toxic than comparable platinum drugs. The treatment inhibited tumor cell proliferation and induced tumor cell apoptosis through ROS generation and JNK/p53-mediated Fas and Bax upregulation. We suggest that diplatin is a promising candidate for clinical development. Diplatin has entered Phase I clinical trials under the regulation of China Food and Drug Administration in 2018.

## Data Availability

The raw data supporting the conclusions of this manuscript will be made available by the authors, without undue reservation, to any qualified researcher.

## Ethics Statement

This study was carried out in accordance with the recommendations of Guide for the Care and Use of Laboratory Animals of Zhejiang University, Zhejiang University Animal Care and Use Committee. The protocol was approved by the Zhejiang University Animal Care and Use Committee.

## Author Contributions

XL and YLJ designed and performed experiments, as well as analyzed the data. XL also wrote the manuscript. XD, JS, and YCJ assisted in performing some of the animal experiments. EA, JCZ, and JPZ performed some of the *in vitro* experiments, EA also advised and reviewed the manuscript. YL and XC synthesized diplatin and performed solubility assay. FW performed toxicology experiments. QX and YX designed the study, supervised the overall project, and wrote the manuscript.

## Funding

This work was supported by grants from the National Natural Science Foundation of China (No. 81573439, No. 81373224, and No. 81803544) and Zhejiang Provincial Public Welfare Technology Application Research Project (LYY18H310005).

## Conflict of Interest Statement

YLJ, XD, JS, and YCJ were employed by Breath Smooth Biotech Hangzhou Co., LTD. YL and XC were employed by Beijing Shuobai Pharmaceutical Co., LTD. FW was employed by Joinn Laboratories, BAD.

## Abbreviations

DDP, cisplatin; CBP, carboplatin; JNK, Jun N-terminal kinse; MTT, 3-(4,5-dimethylthiazol-2-yl)-2,5-diphenyl-2H-tetrazolium-bromide; FBS, fetal bovine serum; ROS, reactive oxygen species; NAC, N-acetylcysteine; PBS, phosphate-buffered saline; DCFH-DA, 2′, 7′-dichlorofluorescein; DMSO, dimethylsulfoxide; EdU, 5-ethynyl-2′-deoxyuridine; LD_50_, median lethal dose; ED_50_, median effective dose; i.v., intravenous; s.c., subcutaneous.
