# Corrigendum: Diplatin, a Novel and Low-Toxicity Anti-Lung Cancer Platinum Complex, Activation of Cell Death in Tumors *via* a ROS/JNK/p53-Dependent Pathway, and a Low Rate of Acquired Treatment Resistance

**DOI:** 10.3389/fphar.2022.890886

**Published:** 2022-04-28

**Authors:** Xixi Lin, Yongliang Jia, Xinwei Dong, Jian Shen, Yachao Jin, Yanyou Li, Fang Wang, Eitan Anenberg, Jiancang Zhou, Jianping Zhu, Xiaoping Chen, Qiangmin Xie, Yicheng Xie

**Affiliations:** ^1^ Children’s Hospital, Zhejiang University School of Medicine, Hangzhou, China; ^2^ Zhejiang Respiratory Drugs Research Laboratory of Food and Drug Administration of China, Zhejiang University School of Medicine, Hangzhou, China; ^3^ Breath Smooth Biotech Hangzhou Co., LTD, Hangzhou, China; ^4^ Beijing Shuobai Pharmaceutical Co., LTD, Beijing, China; ^5^ Joinn Laboratories, BAD, Beijing, China; ^6^ Sir Run Run Shaw Hospital, Zhejiang University School of Medicine, Hangzhou, China

**Keywords:** lung cancer, Platinum complex, Water solubility, ROS/p53 pathway, cisplatin resistance

In the original article, there was a mistake in Figure 3H as published. An incorrect image for the p53 measurement in A549 was shown. The corrected Figure 3H appears below.

**FIGURE 3:**
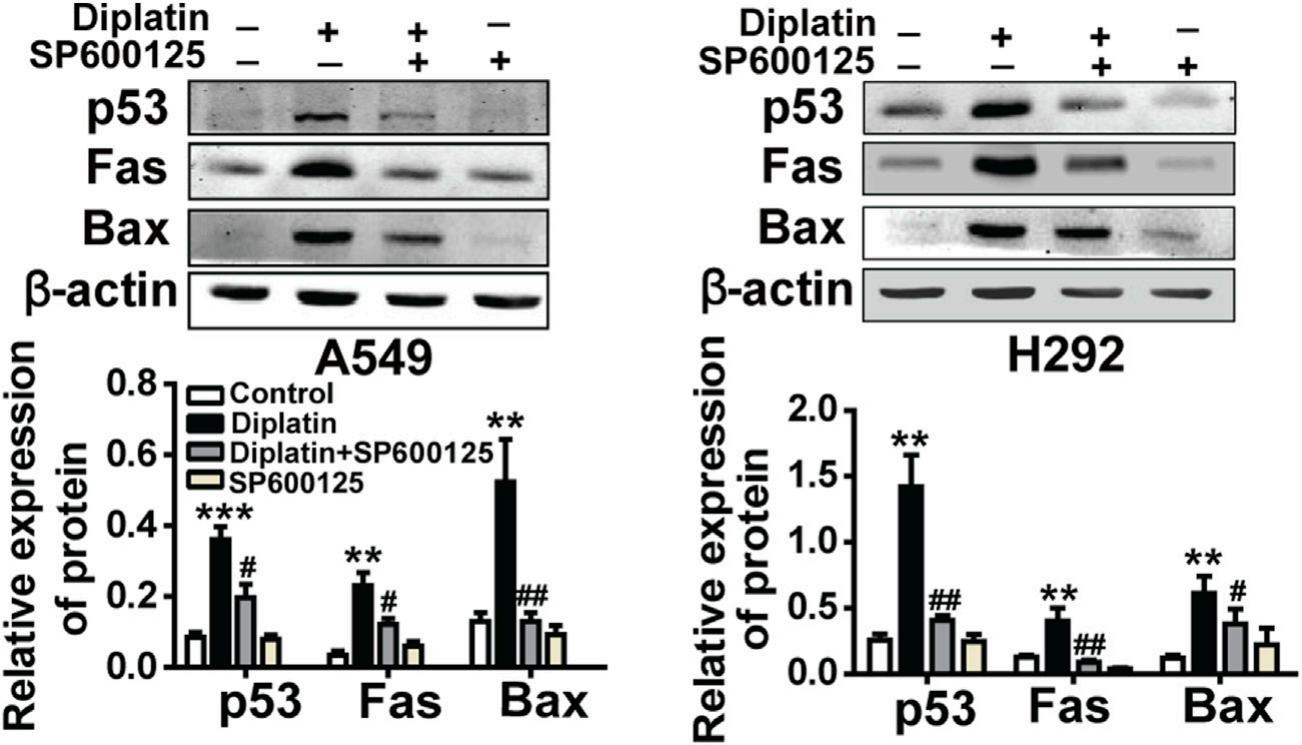
JNK/p53-mediated pathway is involved in diplatin-induced apoptosis of tumor cells. **(H)** Pretreatment (0.5 h) with 10 μM JNK inhibitor (SP600125) suppresses the 48 h time point 25 μM diplatin-induced p53, Fas, and Bax protein upregulation (*n* = 4 per group). The data are presented as mean ± SEM from three independent experiments, one-way ANOVA followed by the Student-Newman-Keuls test. Statistical significance is indicated by *p < 0.05, **p < 0.01, and ***p < 0.001 for comparison with control and #*p* < 0.05, ##*p* < 0.01 for comparison with the diplatin-treated group.

The authors apologize for this error and state that this does not change the scientific conclusions of the article in any way. The original article has been updated.

