# Supplementary material for: Diplatin, a Novel and Low-Toxicity Anti-Lung Cancer Platinum Complex, Activation of Cell Death in Tumors via a ROS/JNK/p53-Dependent Pathway, and a Low Rate of Acquired Treatment Resistance
Source: Front Pharmacol. 2019 Sep 11;10:982. doi: 10.3389/fphar.2019.00982 (PMC6749073; doi:10.3389/fphar.2019.00982)
Supplement: Supplementary file 1 [file DataSheet_1.docx]

**Supplementary Data**

**Supplement 1. Diplatin exerts favorable effects on the suppression of the other tumor cells.**

To evaluate the effects of diplatin on the other tumor cells, we determined the effects of diplatin, DDP and CBP on the viability of another 10 cancer cell lines after 48 h treatment (Supplementary Table 1). We found diplatin suppressed the viability of the tumor cells more than that was observed with CBP treatment. Particularly, diplatin had a strong potency in killing gastric cancer cells, which was equivalent to the effects of DDP and nearly 5-fold more potent than CBP. These results demonstrate that diplatin also had favorable effects on the suppression of the other tumor cells other than the lung tumor cells.

**
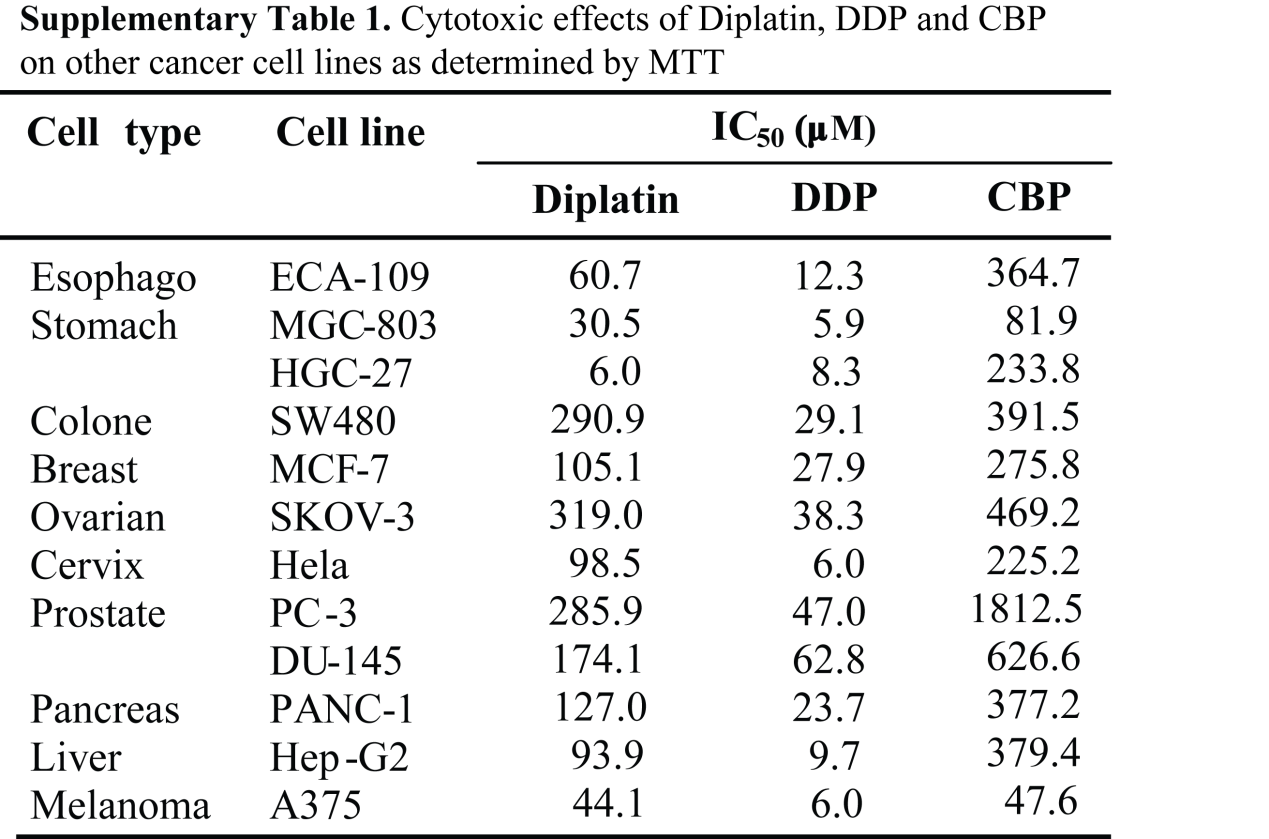
**

**Supplement 2. Pharmacokinetic and toxicokinetic characterization of diplatin treatment in dogs indicates its non-inferior pharmacokinetics and favorable safety**

Since investigation of pharmacokinetic and toxicokinetic characterization requires frequent blood sampling, we employed Beagle dogs to evaluate diplatin (Supplementary Table 2). A single dose of diplatin administration at 20, and 40 mg/kg showed a longer half-life (T_1/2_) compared to the ones treated with 10 mg/kg. Maximum platinum levels in the plasma all reached in 0.33 h. The C_max_ ratio and AUC _(0-144 h)_ ratio were more in the high dose group. Twenty-one days after being given the high dose of repetitive treatment of diplatin, the dogs showed a similar T_1/2_, higher AUC _(0-144 h)_ and AUC_INF_ compared to the DDP treatment. Furthermore, AI was employed to evaluate the accumulated toxicity of the two complexes. We found DDP treated group had nearly two-fold higher AI value than the group treated with diplatin at 10, 20 or 40 mg/kg. Our data suggest that diplatin exhibited non-inferior pharmacokinetics but a lower toxicity compared to the DDP treatment in dogs.

**
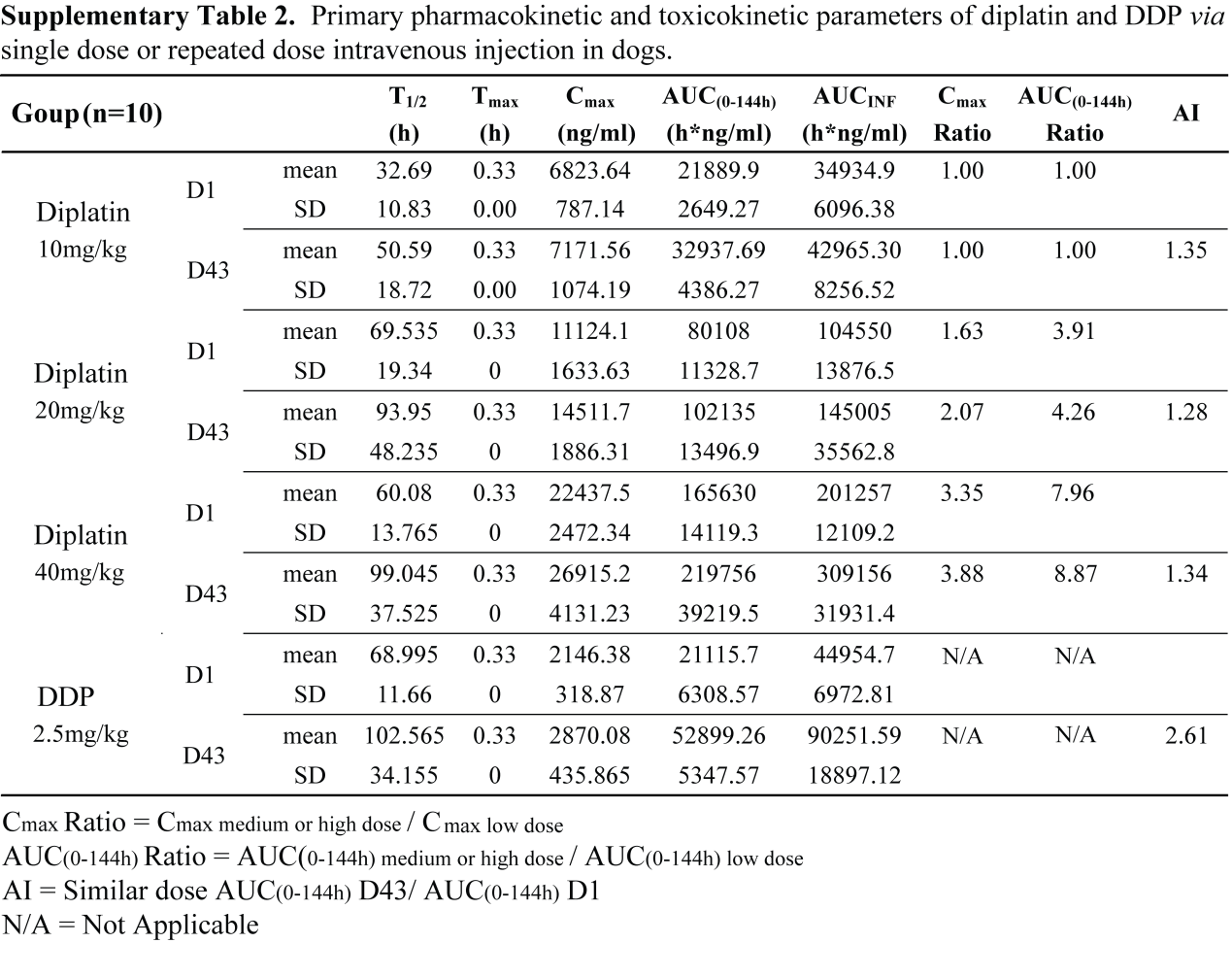
**

**Supplement 3. Diplatin treatment exerts little effects on the** **appearance of mice**

Thirty-two ICR mice were randomized into 4 groups (n=8, half male and half female), which were injected intravenously in the tail vein with diplatin at the doses of 60, 120 or 240 mg/kg twice a week for 4 weeks. The animals were observed daily for any evident symptoms of sickness. Changes in their appearance (e.g., kyphosis and altered grooming), behavior (altered nesting) and activity (altered exploring) were monitored for general neurotoxicity assessment. We found diplatin chronic treatment was generally well tolerated by the animals, the general behaviors were not significantly changed, even at the dose of 240 mg/kg, and only approximately 20% of animals showed signs of mild kyphosis and piloerection (Supplementary Table 3).


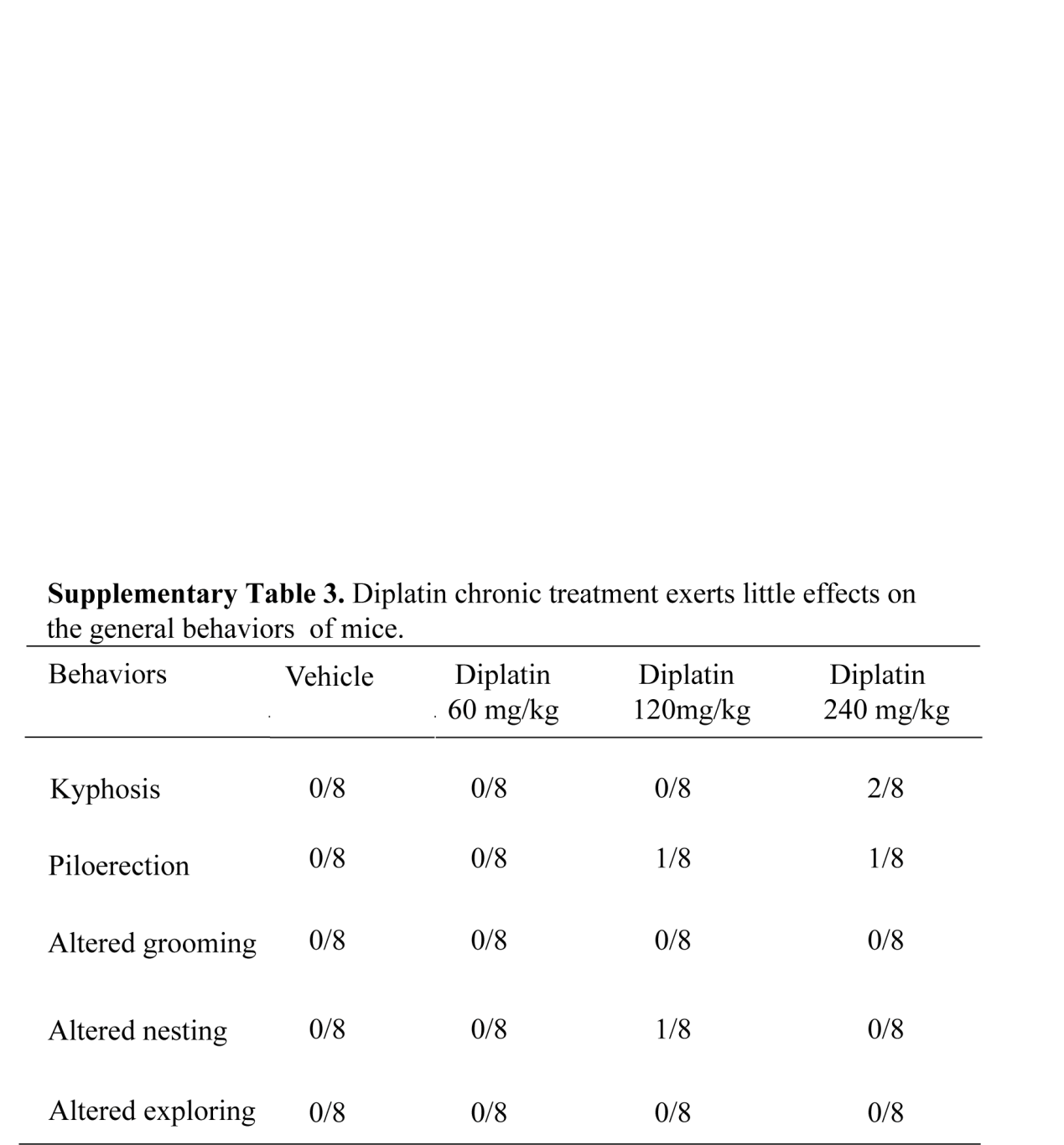


**Supplement 4. Diplatin treatment causes nephrotoxicity in rats.**

To further study the possible nephrotoxicity that caused by diplatin, we detected renal pathology changes of the rats after various concentrations (7.5 mg/kg, 15 mg/kg, and 30 mg/kg) of diplatin administration (Supplementary Table 4). Total 30 rats were employed in the experiments, 20 rats were euthanized with isoflurane  3 days after the last administration, and the kidneys were excised and examined by the H&E staining, and the remaining 10 rats were handled after the recovery period (two weeks after the last administration). We found drug-related renal changes were appeared in the rats, especially in the high dose group (30 mg/kg). Of all the changes, the renal tubular epithelium denaturation/regeneration and tubular dilation are the most obvious, which are early appeared after 7.5 mg/kg treatment at both last administration and during recovery period. The results suggest that nephrotoxicity existed in the diplatin treated rats.


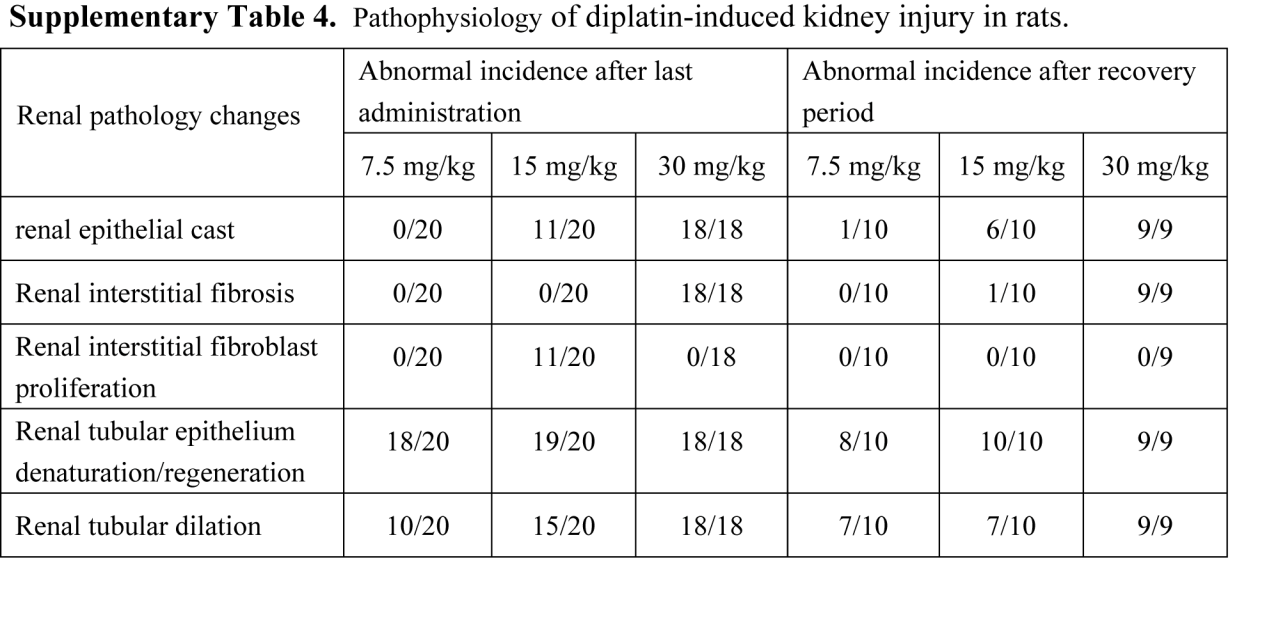


**Supplement 5. Diplatin treatment shows no obvious adverse effects on the kidney.**

To measure diplatin accumulation in tissues, A549 xenograft nude mice were intravenously administrated with 120 mg/kg diplatin (equivalent to Pt 36.78 mg/kg). At each time point of 10 min, 2 h, 6 h and 24 h after administration, 6 mice were sacrificed. The tumors, organ/tissue, and blood were immediately collected. Then, Pt concentration was measured by atomic absorption spectrometry, counting the weight/volume of the tumors, tissues or organs/blood. As shown in Supplementary Figure 1, we found large amounts of diplatin in the kidney, compared to the other organs (Supplementary Figure 1A). Pt accumulation was 2.2 times higher in the kidney in the diplatin treated group at 6 h after administration, compared to CBP given at 70 mg/kg (equivalent Pt dose to 120 mg/kg diplatin, Supplementary Figure 1B). There was higher AUC in the kidney 6 h after the 120 mg/kg diplatin treatment compared to that in the other organs or in the blood (Supplementary Figure 1C).


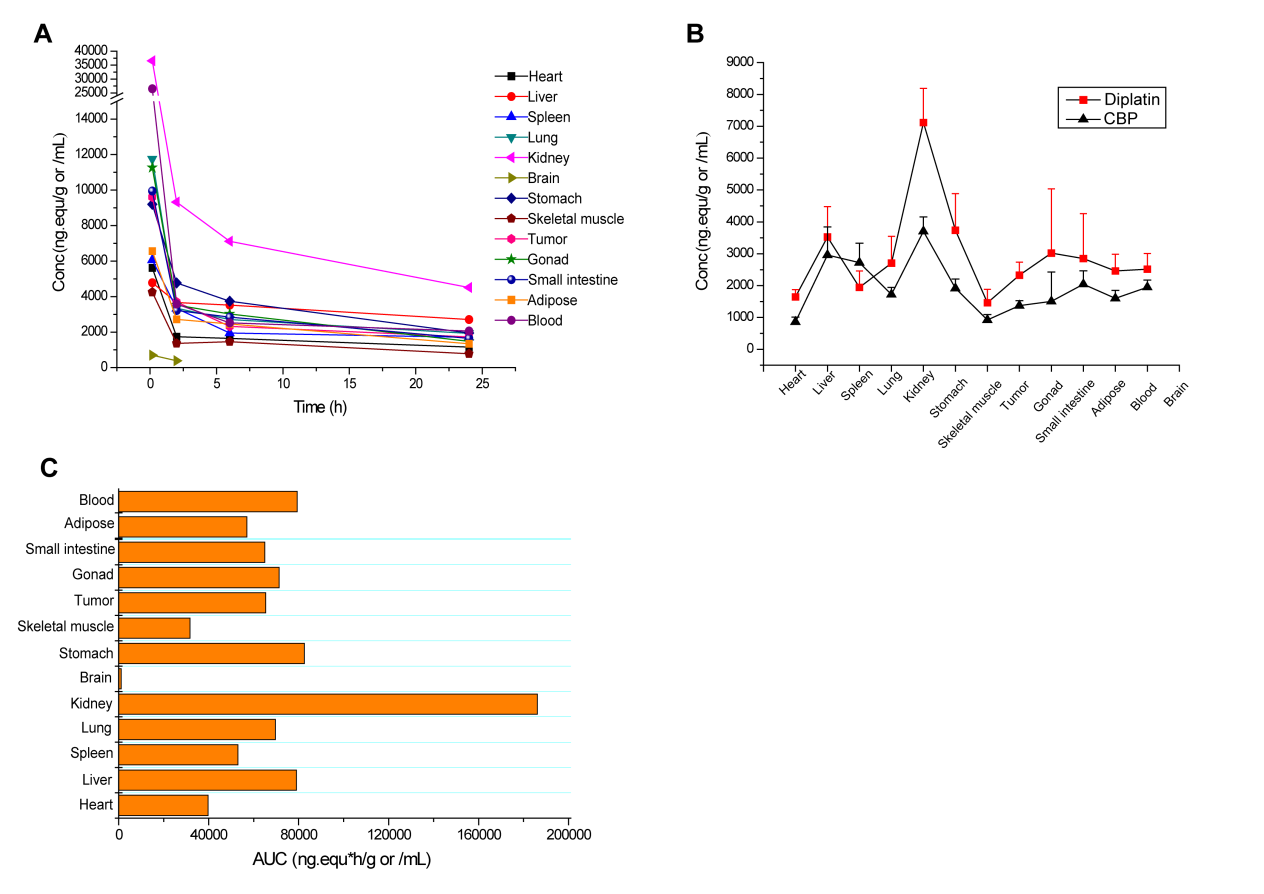


**sFigure 1. The bio-distribution of diplatin in mice.** (A) A time profile of diplatin tissue distribution measured as Pt concentration after 120 mg/kg i.v. administration (*n*=6). (B) Tissue distribution of 120 mg/kg diplatin and 70 mg.kg CBP measured as Pt concentration at 6 h after i.v. administration (n=6). (C) AUC values of various tissues at 6 h after a single dose of 120 mg/kg diplatin i.v. administration (n=6).

**Supplement 6. Diplatin treatment induces cell apoptosis through the p53- dependent pathway in LTEP-A-2 xenograft lung tumor.**

The antitumor effects of diplatin on LTEP-A-2 xenograft tumors were evaluated. The diplatin-treated group showed a significant inhibition on xenograft tumor volume and weight. As observed in Supplementary Figure 2A, diplatin dose-dependently inhibited the growth of the LTEP-A-2 xenograft tumor. At the dose of 120 mg/kg, the inhibitory effects of diplatin on xenograft tumor volume and weight were comparable to the effects of DDP at 6 mg/kg and CBP at 60 mg/kg.

To elucidate possible anti-tumor mechanisms, we examined whether the tumor inhibition by diplatin was resulted from its induced cell apoptosis, visualized by TUNEL staining (Supplementary Figure 2B). Diplatin (120 mg/kg) treated LTEP-A-2 tumors exhibited a markedly increased apoptosis, which was comparable to the effects of DDP (6 mg/kg) and CBP (60 mg/kg) treatment. The effects of diplatin on tumor angiogenesis was investigated by immunolabeling VEGF, and the effect on tumor cell apoptosis was measured by immunolabeling p53, Bax in tumor sections. As shown in Supplementary Figure 2C, the diplatin-treated group (at 120 mg/kg) showed lower expression of VEGF (*P*<0.001) compared with the control, indicating less tumor angiogenesis. Moreover, the diplatin treatment significantly enhanced p53 and Bax expression in the LTEP-A-2 tumor sections.


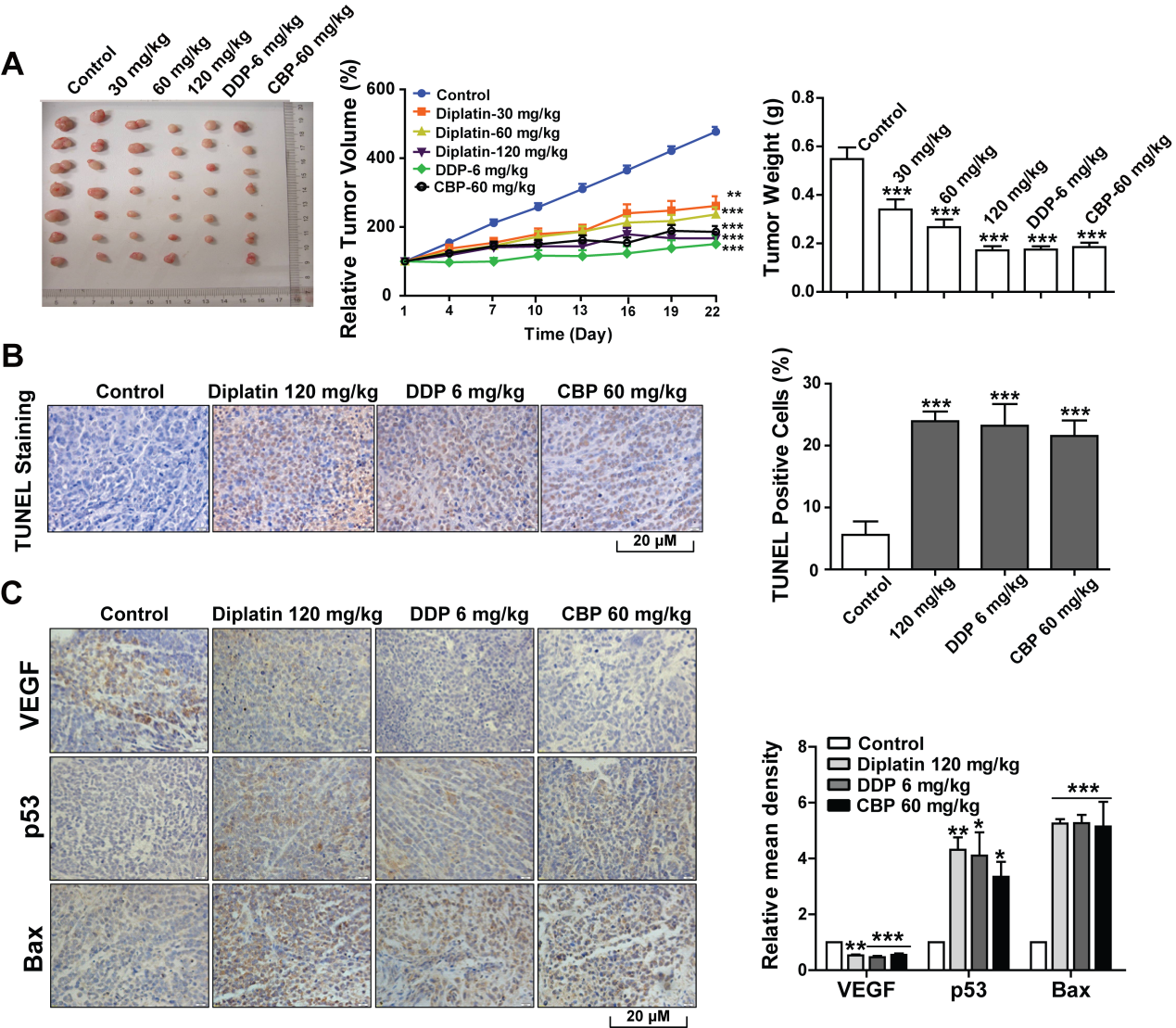


**sFigure 2. Diplatin treatment exhibits potent anti-tumor activities against LTEP-A-2 xenograft lung tumors.** (A) Diplatin dose-dependently inhibits LTEP-A-2 xenograft tumor volume and weight compared with the control. Diplatin at 120 mg/kg shows a comparative inhibition of LTEP-A-2 xenograft tumor as DDP at 6 mg/kg and CBP at 60 mg/kg treatment. The data represent the mean ± S.E.M (n = 14). ***P*<0.01 and ****P*<0.001 versus the control. (B) Diplatin (120 mg/kg) considerately enhanced apoptosis in LTEP-A-2 tumor sections as assessed by TUNEL staining. Positive cells are in dark brown color. Representative TUNEL images for cell apoptosis are presented (scale bar 20 μm). Quantitation of tumor apoptosis is expressed as the percentage of apoptotic cells (apoptotic index). Results are presented as the mean ± S.E.M from three independent experiments. ****P*<0.001 versus the control. (C) Immunohistochemical staining of VEGF, p53 and Bax expression in the xenograft LTEP-A-2 lung tumor tissues following treatment are shown. The illustrated images are the representatives of three independent experiments (scale bar 20 μm). **P*<0.05, ***P*<0.01 and ****P*<0.001 compared with the control.

**Supplement 7. Diplatin treatment causes cell in G2/M phase in LTEP-A-2 cells, but not in H460 cells.**

Flow cytometry was further employed to evaluate the effects of diplatin on the distribution of cells in LTEP-A-2 and H460 cells (Supplementary Figure 3). DDP and CBP treatment induced cell cycle arrest in S phase. Diplatin at the dose of 50 uM caused a significant accumulation of cells in G2/M phase in LTEP-A-2 cells, whereas, like in H292 cells, diplatin treatment failed to arrest the cells in the G2/M phase of the cell cycle in H460 cells.

**
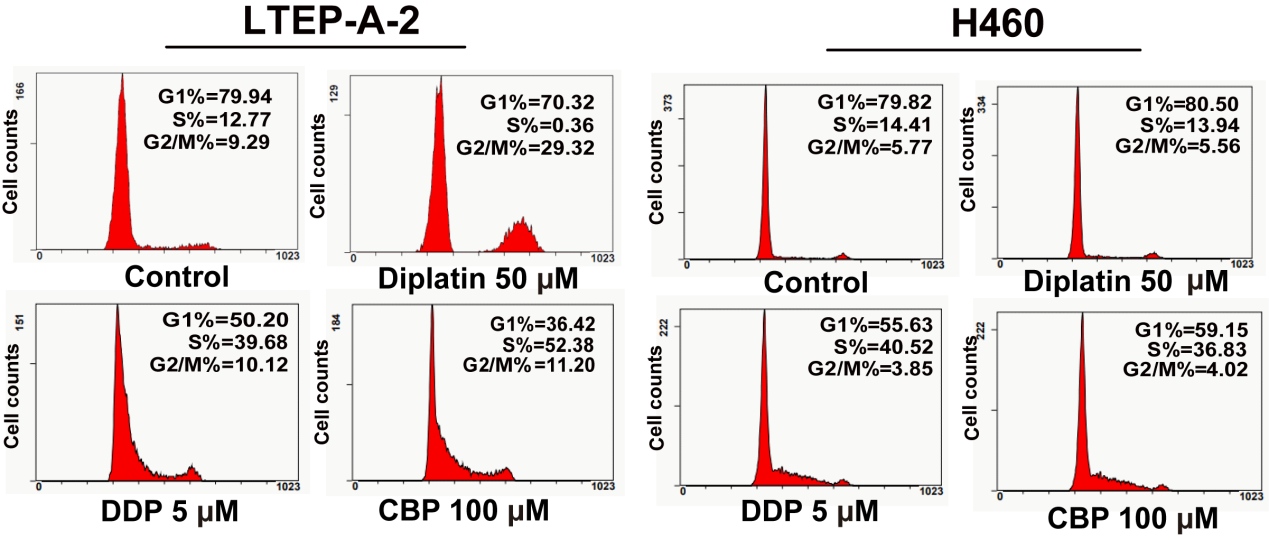
**

**sFigure 3.** Effects of diplatin (50 µM), DDP (5 µM), and CBP (100 µM) on cell cycle in LTEP-A-2 and H460 cells. Representative DNA fluorescence histograms of propidium iodide (PI)-stained cells are presented.

**Supplement 8. Diplatin treatment induces** **JNK activation in the p53-mediated apoptosis in the lung tumor cells.**

To explore the molecular mechanisms underlying the regulation of lung tumor cells apoptosis by the diplatin-induced p53 expression, we evaluated whether MAPK signaling pathway was implicated in the p53-dependent apoptosis in response to diplatin administration. Our data suggest that diplatin (10 μM, 25 μM and 50 μM), DDP (5 μM) or CBP (100 μM) failed to induce Erk1/2 and p38 phosphorylation in A549 and H292 cells (Supplementary Figure 3A). Furthermore, JNK inhibition by SP600125, other than p38 inhibitor (SB203580), MEK inhibitor (U0126) or PI3K inhibitor (LY294002), significantly suppressed the diplatin-induced p53, Fas and Bax expression (Supplementary Figure 3B), demonstrating an important role of JNK activation in the p53-mediated apoptosis.

**
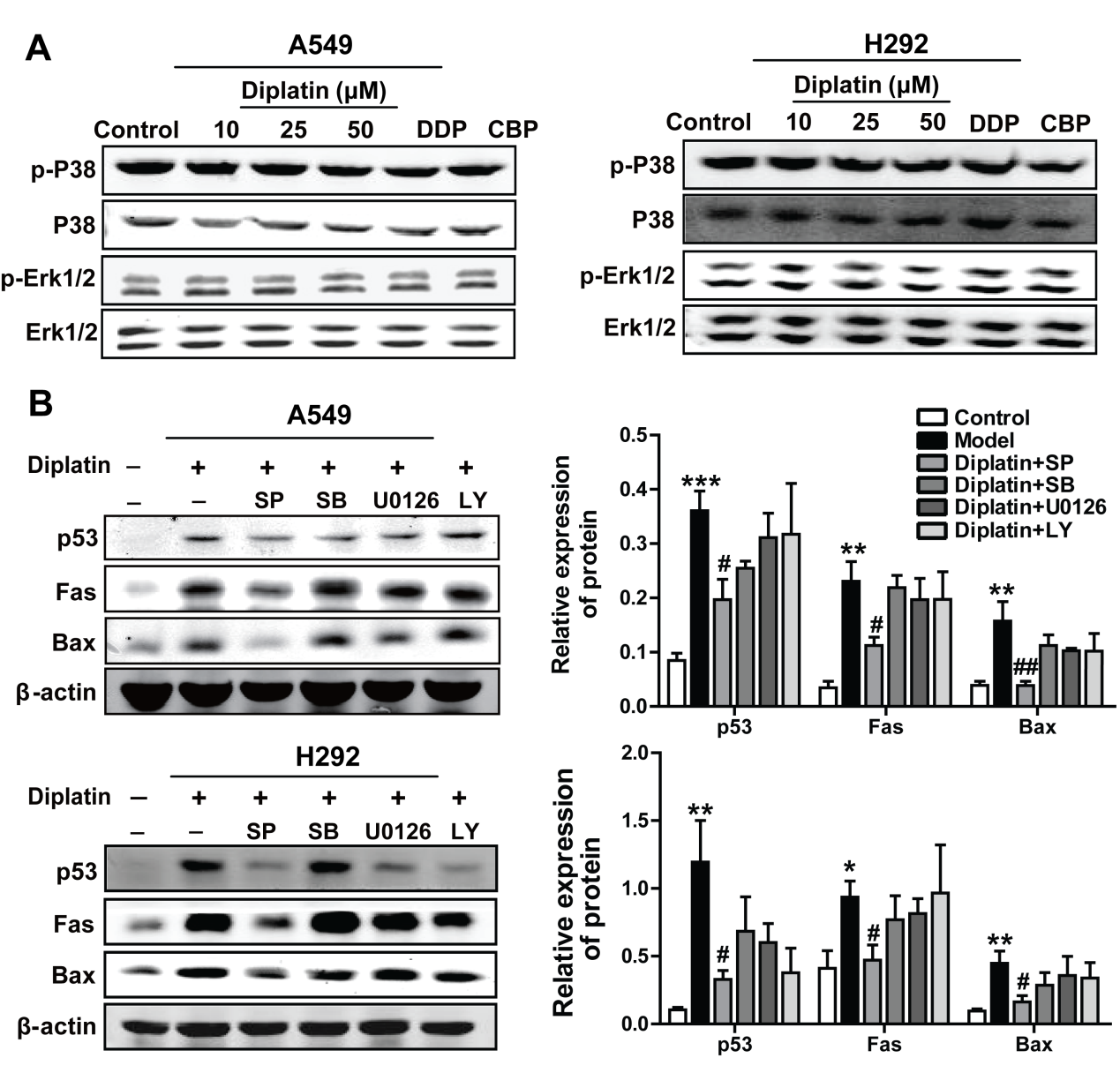
**

**sFigure 4. Diplatin treatment induces JNK activation, other than Erk1/2 or p38, in the p53- mediated apoptosis in the lung tumor cells.** (A) Diplatin at various concentrations, DDP at 5 μM and CBP at 100 μM does not activate Erk1/2 or p38 in A549 and H292 cells. (B) The JNK inhibitor SP600125 (SP), other than the p38 inhibitor (SB203580, SB), the MEK inhibitor (U0126) or the PI3K inhibitor (LY294002, LY), suppresses the diplatin-induced p53, Fas and Bax up-regulation. The data represent the mean ± S.E.M from three independent experiments. **P*<0.05, ***P*<0.01，****P*<0.001 compared with the control, ^#^*P*<0.05, ^##^*P*<0.01 compared with the diplatin-treated group.
